# A New School Physiology

**Published:** 1883-09

**Authors:** 


**A New School Physiology.** By RICHARD J. DUNGLISON, A. M, M. D., author of Practitioner's Reference Book. Editor of Dunglison's Medical Dictionary, etc. Illustrated with 117 engravings. Philadelphia: Porter & Coates.

That a knowledge of Physiology is important and desirable for young people to have, is an assertion which no one will dispute. A good text-book for public schools has been needed, and we think that this work of Dr. Dunglison's fairly meets the demand. We have spoken favorably of this work, on its first appearance, and we again commend it.

